# Development of a Single-Sided Nuclear Magnetic Resonance Scanner for the In Vivo Quantification of Live Cattle Marbling

**DOI:** 10.1007/s00723-015-0657-4

**Published:** 2015-03-18

**Authors:** Yoshito Nakashima

**Affiliations:** National Institute of Advanced Industrial Science and Technology (AIST), Central 7, 1-1-1 Higashi, Tsukuba, Ibaraki 305-8567 Japan

## Abstract

Non-invasive in vivo marbling quantification helps owners to choose the optimum nutritional management for growing cattle and buyers to more precisely evaluate grown cattle at auctions. When using time-domain proton nuclear magnetic resonance (NMR) relaxometry, it is possible to quantify muscle and fat separately by taking advantage of the difference in the spin–spin relaxation time (*T*2) between water molecules in muscles and fat molecules, which would contribute to the non-invasive and objective determination of marbling scores. With this in mind, we developed a prototype NMR scanner (4.1 MHz for protons) using an original single-sided magnetic circuit and a plane radio frequency (RF) coil for use in the non-invasive quantification of water and fat in live cattle. The sensed region of the developed scanner is compact and almost cubical (19 × 19 × 16 mm^3^) while the investigation depth (the distance from the RF coil to the center of the sensed region) has been lengthened to 30 mm, which is sufficient for the in vivo trapezius muscle measurement of live cattle. Measurements of 17 samples of beef meat blocks kept at 39 °C were taken in a laboratory to successfully obtain the calibration lines used to convert the NMR signals into water and fat weight fractions at correlation coefficients in excess of 0.9. We also showed that each meat sample could be measured in about 10 s with a measurement error as small as approximately 10 wt%. Accordingly, we believe that our prototype scanner would be useful for in vivo marbling measurements of live cattle trapezius muscles.

**Electronic supplementary material:**

The online version of this article (doi:10.1007/s00723-015-0657-4) contains supplementary material, which is available to authorized users.

## Introduction

Intramuscular fat content, commonly referred to as marbling, is an important factor when determining beef meat quality [1]. The nutritional management (growth path) of cattle is an important factor controlling the marbling degree [2–4]. Thus, non-invasive monitoring of the marbling degree of live cattle from birth to auction is desirable for owners to evaluate the effects of growth path on marbling, and for buyers to more precisely evaluate the meat quality of grown cattle. One of the possible solutions for in vivo marbling monitoring is the use of time-domain (not imaging) low-field nuclear magnetic resonance (NMR) surface scanners using single- or one-sided permanent magnets [5, 6], which are both portable and inexpensive. The NMR scanners enable users to quantify water and fat content separately by taking advantage of the difference in the proton spin–spin relaxation time (*T*2) between water molecules in muscles and fat molecules [7] (see also Table 1). Unfortunately, the investigation depth (distance from the coil to the center of the sensed region) of the single-sided magnets previously developed for fat/water measurements in food (e.g., beef, fish, and dairy product) was in the order of several millimeters [8–12] due to the small dimensions of the magnets used. Such a depth of investigation is too shallow to probe the marbled muscle beneath the thick subcutaneous fat and skin of live cattle.

**Table 1:** Typical properties of beef meat samples analyzed by proton NMR at 39 °C

Sample	*T*1 at 4.1 MHz^a^ (ms)	*T*2 at 4.1 MHz^b^ (ms)	Self-diffusivity at 20 MHz^c^ (m^2^/s)
A (fat)	114	156	2.4 × 10^−11^
E (lean meat)	287	68	1.9 × 10^−9^

*T*1 and *T*2 refer to the spin–lattice relaxation time and spin–spin relaxation time, respectively. See electronic supplementary material (Table ESM_1) for sample details

^a^By the saturation recovery method

^b^By the PAPS CPMG method

^c^By the pulsed-gradient spin-echo method with a gradient pulse interval of 28 ms using the apparatus described in [29]

In response to this issue, we have developed a prototype single-sided NMR scanner using a permanent magnet large enough to provide an investigation depth of 30 mm, which is sufficient to probe the trapezius muscle of live cattle. For our device, special care was taken to produce a large homogeneous magnetic field remote from the magnet surface, and to reduce undesirable coherent/incoherent radio frequency (RF) noise. In the present study, the technical aspects of the magnetic circuit, RF coil, and the pulse sequence used are disclosed to facilitate the use of our single-sided NMR for in vivo objective marbling measurements. The weight fractions of water and fat in beef meat block samples were also measured in our laboratory at 39 °C to demonstrate the time requirements and accuracy of the measurement process.

## Single-Sided NMR Surface Scanner

The beef marbling score is conventionally determined using the longissimus or rib eye muscle located about 10 cm beneath the cattle’s skin. However, it would be very expensive and dangerous to construct a permanent magnet large enough to probe so deeply into live cattle. In contrast, the trapezius muscle, which is located at about 3 cm from the skin [30, 31], is significantly more accessible than the longissimus muscle. Fortunately, a number of studies have revealed a certain degree of correlation between the trapezius and longissimus muscles with respect to marbling degree [13, 14]. Thus, the trapezius muscle can be considered a potential substitute for the longissimus muscle when evaluating marbling degree. With these thoughts in mind, we developed our NMR surface scanner for in vivo measurements of the trapezius muscle, thus allowing us to reduce the magnet size and cost.

**Fig. 1:**
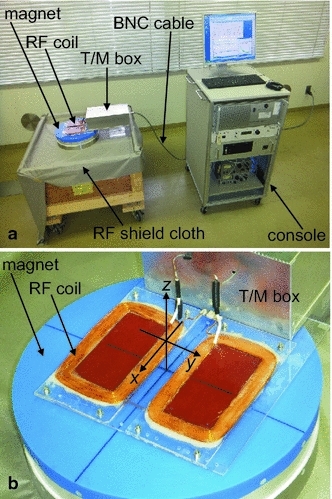
Photos of the NMR surface scanner apparatus. **a** Whole system. **b** Close-up of the magnet and RF coil. The RF coil and T/M box are connected to the console via a double-shielded BNC cable. The magnet is placed on the wooden box used for transporting it. The diameter of the magnet’s blue plastic cover is 313 mm. The origin of the *x*–*y*–*z* coordinate system is located 47 mm above the blue cover and 30 mm above the RF coil. The *z*-axis of the *x*–*y*–*z* system coincides with the cylindrical magnet axis

The developed NMR scanner system is shown in Fig. 1. The system consists of a console (MRTechnology Inc., Tsukuba, Japan), a Nd–Fe–B magnet, an RF coil, and a tuning and matching (T/M) box connected with a Bayonet Neill–Concelman (BNC) cable. Thanks to the single-sided magnet geometry, the three-dimensional space shown as *z* > −30 mm in Fig. 1b is available for free space use when probing the surface of large objects such as cattle (although the T/M box is located at *z* > −30 mm for the experimental convenience in Fig. 1b, it can readily be moved to *z* < −30 mm).

**Fig. 2:**
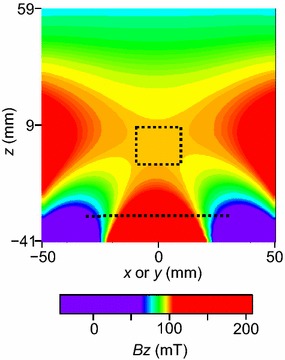
Axisymmetric distribution of the *z*-component of the magnetic flux density vector (*B*_*z*_) above the end face of the cylindrical magnet as measured by a Tesla meter with a Hall probe. The RF coil position (*z* = −30 mm) and sensed region (19 × 16 mm^2^) defined by Fig. 3 are indicated by the *dotted line* and *rectangle*, respectively

**Fig. 3:**
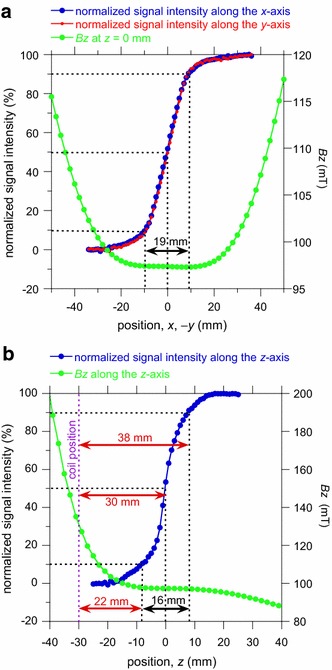
Position of the sensed region determined using the spatial distribution of the normalized NMR signal intensity that was obtained by the rubber-sheet experiments. Regarding the normalized signal intensity, the corresponding horizontal axis is the front position of the removal of the stacked sheets (i.e., position of the sheet *colored in green* in Fig. ESM_5). The *B*_*z*_ profile obtained from Fig. 2 is superimposed. **a** Sensitivity distribution along the *x*- and *y*-axes. **b** Sensitivity distribution along the *z*-axis. The sensed region is indicated by a *black arrow*. The origin of the *z*-axis (i.e., *z* = 0 mm) coincides with the center of the sensed region where the normalized signal intensity is 50 %

A brief structural overview of the single-sided magnet shown in Fig. 1 is included in the Electronic Supplementary Material (Fig. ESM_1). The basic idea, which involves employing a cylindrical magnet with another centered magnet, was taken from Fukushima and Jackson [15]. This magnetic circuit yields a large sweet spot where all first-order spatial derivatives of the magnetic field are zero at locations remote from the magnet end face. The detailed spatial distribution of the magnetic flux density around the sweet spot is shown, along with the position of the sensed region, in Figs. 2 and 3. The magnetic flux density is 97 mT at the center of the sensed region (i.e., at *x* = *y* = *z* = 0 mm), corresponding to the Larmor frequency of 4.1 MHz for protons. The advantage of a magnet with a sweet spot over a magnet without one [16] is that the time required for the NMR data acquisition is shortened because (a) the NMR signal intensity is larger due to the large volume of the sweet spot, and (b) the noise intensity is suppressed by narrowing the RF receiver bandwidth. This time shortening is essential for the measurement of the live cattle because it is difficult to get a live animal to remain still long enough to perform long-time signal stacking unless sedative/anesthetic is used. It should be also noted that the sweet spot shown in Figs. 2 and 3 is compact (i.e., nearly cubical) in dimension, which is an important feature for precisely identifying the meat portion from where the NMR signal arises. In contrast, single-sided NMR well-logging tools have very elongated sensed regions (several tens of centimeters along the longitudinal direction of the tools; see ref. [5]), which obscure the location of the signal source due to undesirable averaging.

The investigation depth of the RF coil developed in our previous study [17, 18] was ≈10 mm, and thus did not allow us to probe the trapezius muscle, which is approximately 30 mm beneath the cattle’s skin. Therefore, a new RF coil was fabricated for the present study to achieve the necessary investigation depth (i.e., 30 mm). Details regarding the new RF circuit are shown in Fig. ESM_2. When compared to the apparatus used in our previous study, the gap between the two subunits was broadened from 0 to 22 mm to effectively excite protons located 30 mm away from the coil surface. The capacitors in the T/M box were also replaced with those having larger withstanding voltages. A copper foil with slits was fabricated and inserted between the RF coil and magnet to reduce the undesirable eddy currents induced by the RF pulses.

The NMR measurement is adversely affected by the ringing noise caused by mechanical coil vibrations when strong RF pulses are produced. Simple signal stacking does not provide an effective countermeasure because the noise is synchronized with the RF pulse timing. Therefore, a modified Carr-Purcell-Meiboom-Gill (CPMG) pulse sequence, specifically, a phase-alternated pair stacking (PAPS) CPMG pulse sequence [19], was used in the present study to reduce such coherent noise. The principles of the PAPS CPMG sequence are shown in Fig. ESM_3, and a successful example is shown in Fig. ESM_4.

The NMR coil also receives incoherent (i.e., random) noise from the outside environment. In an effort to reduce such random noise, high-performance RF shield cloth and double-shielded BNC cables, which were not used in our previous study [17, 18], were employed (Figs. 1 and ESM_7).

## Experimental

Two sets of experiments were performed: one experiment set using silicon rubber sheets and the other using beef meat blocks. Since soft rubber sheets have relatively long *T*2 values, they are often used to confirm the performance of the single-sided NMR [16]. In our experiments, silicon rubber sheets stacked in the *x*-, *y*-, and *z*-directions were used to probe the position and dimensions of the sensed region. The obtained sum of echoes as NMR signal intensity was normalized and its dependence on the number of the stacked rubber sheets was examined. Other experimental details are shown in Fig. ESM_5.

In the next set of experiments, the weight fractions of water and fat in beef meat block samples maintained at 39 °C were measured to demonstrate the applicability of our NMR system to live cattle. First, 17 boneless beef blocks were purchased from local meat stores. The details of the meat samples are listed in Table ESM_1. The samples were cut into smaller blocks (typically ≈8^3^ cm^3^ in size), and then vacuum sealed in thin plastic films to prevent undesired water evaporation and fat alternations during our experiments. Since the rectal temperature of live cattle is approximately 39 °C [20], the meat samples in this study were first suspended in a 39 °C water bath for a few hours, placed on the RF coil (Fig. ESM_6), covered with RF shield cloth (Fig. ESM_7), and then quickly measured via the PAPS CPMG method before the temperature of the sensed region deep in the sample (Fig. ESM_8) could change significantly. The parameters of the PAPS CPMG sequence were as follows: duration of the 90° and 180° pulses, 0.2 ms; echo spacing, 0.5 ms; A/D convertor sampling rate, 0.005 ms; number of data sampling points per echo, 40; sampling window length, 0.195 ms; sequence repetition time, 3000 ms (i.e., *T*1 full relaxation condition); number of echoes, 1200; number of signal stacking, 4, 8, 16, and 32 (i.e., number of PAPS pairs, 2, 4, 8, and 16, respectively); RF receiver bandwidth, ±100 kHz. The resultant Q (quality factor) value of the RF coil with a meat block sample was approximately 40.

After the NMR measurements, a small piece of meat (25 × 25 × 20 mm^3^) was cut from each sample, taking care to include the sensed region of 19 × 19 × 16 mm^3^ (Fig. ESM_8). These meat pieces were then analyzed by conventional chemical analysis techniques to determine their true contents (fat by the Soxhlet extraction method and water by the air oven method). By comparing the results by NMR and chemical analysis, the accuracy of the NMR method could then be evaluated. The dependence of the accuracy on the measurement time (i.e., number of signal stacking) was also examined.

The process used to distinguish the NMR signals derived from fat and those derived from muscle will now be explained. We began by assuming that marbled beef is a mechanical mixture of fat and muscle, and that the muscle content can be represented by the water content. Examples for the end-members of the mixture are listed in Table 1, indicating that *T*2 values are significantly different between pure fat and water in lean meat. Then we have

1 $$ f(t) = a_{\text{fat}} \exp ( - t/T2_{\text{fat}} ) + a_{\text{water}} \exp ( - t/T2_{\text{water}} ) $$

where *f* is the PAPS CPMG time-series data, *t* is time, *a*_fat_ is the NMR signal amplitude for fat, *T*2_fat_ is *T*2 for fat, *a*_water_ is the NMR signal amplitude for water, and *T*2_water_ is *T*2 for water. In the present study, *T*2_fat_ was taken to be 141 ms, which is the arithmetic average of the three fat samples A, B, and C (i.e., *T*2 = 156, 140, and 126 ms); *T*2_water_ was taken to be 61 ms, which is the arithmetic average of the three lean meat samples D, E, and F (i.e., *T*2 = 61, 68, and 55 ms).

Detailed study revealed that water molecules in muscle consist of three *T*2 components [21]. Chemical analysis of the fat samples of A, B, and C was performed using gas chromatography. The analysis revealed that the fat samples contain unsaturated fatty acid (e.g., oleic acid) having a low melting point, and saturated fatty acid (e.g., palmitic acid) having a high melting point, at a weight ratio of 57:43. Because the high (or low) melting point yields short (or long) *T*2 values, fat molecules are likely to consist of at least two *T*2 components. To incorporate this multi-component nature of the *T*2 relaxation for muscle and fat, we preliminary tried multi-exponential decomposition [12] of the PAPS CPMG data. However, no significant improvement was obtained compared with the single-*T*2 component model with respect to calibration line accuracy (Nakashima, unpublished data). Therefore, in the present study, the single-*T*2 component model was assumed for both fat and water as represented by Eq. (1).

During actual measurements, the first few PAPS CPMG echoes received are inevitably transient [22] and contaminated with an undesirable ringing noise tail induced from the free induction decay (see Fig. ESM_3). Thus, the first three echoes were discarded, and the remaining 1200 − 3 = 1197 echoes were fitted to Eq. (1) to obtain *a*_fat_ and *a*_water_ by the least squares method. The obtained *a*_fat_ and *a*_water_ values were combined with the results obtained by conventional chemical analysis to construct calibration lines for fat and water.

## Results

The results of the search for the sensed region are shown in Fig. 3, where it can be seen that the monotonic decrease in signal intensity that accompanies a decrease in the number of stacked rubber sheets is clearly depicted. Tentatively, we set the normalized signal intensities of 10 % and 90 % as the sensed region borders and assume the sensitivity center as the 50 % intensity level. As a result, the sensed region is centered at *x* = *y* = *z* = 0 mm, and the dimensions are 19 mm in the *x*- and *y*-directions and 16 mm in the *z*-direction. It should be noted that the sensed region is located within the sweet spot where the first-order derivatives of *B*_*z*_ are zero. The axisymmetric nature of the magnet and *B*_*z*_ (Figs. 2 and ESM_1) is the main reason for the equal sensed region dimensions in the *x*- and *y*-directions. The sensed region covers 22–38 mm, and is centered at 30 mm from the RF coil (Fig. 3b), thereby allowing in vivo measurements of the trapezius muscle located at about 30 mm beneath the cattle’s skin.

**Fig. 4:**
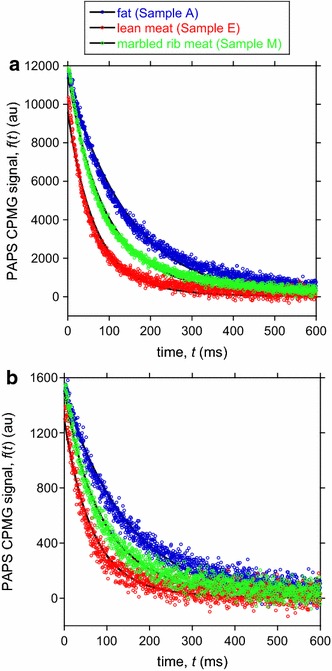
Example of the PAPS CPMG time-series data for three samples (fat, lean meat, and marbled rib meat). See Table ESM_1 for sample notation details. Each data point is the sum of the forty NMR signals acquired at a sampling rate of 0.005 ms in the sampling window (duration of 0.195 ms, see Fig. ESM_4). A model, Eq. (1), fitted by the least squares method is superimposed using a *solid line*. **a** Number of signal stacking = 32. **b** Number of signal stacking = 4

An example for the time-series raw data detected by the RF coil that demonstrates the successful reduction of ringing noise is shown in Fig. ESM_4. Figure 4 depicts examples for the PAPS CPMG time-series data showing the decay of the echo signal intensity averaged over each sampling window. Although the random noise is stronger for the number of signal stacking = 4, the remarkable difference in the decay time constant derived from the difference in the fat–water fraction is depicted with a reasonable signal to noise (S/N) ratio in both Fig. 4a, b. As expected, Sample M (mixture of fat of 34.0 wt% and water of 51.2 wt%) decays with an intermediate time constant of the end-member Samples A (fat 91.7 wt%, water 7.2 wt%) and E (fat 4.3 wt%, water 72.2 wt%). The reasonable fit of Eq. (1) to all obtained data justifies our simple model, which assumes a single decay component for each end-member.

**Fig. 5:**
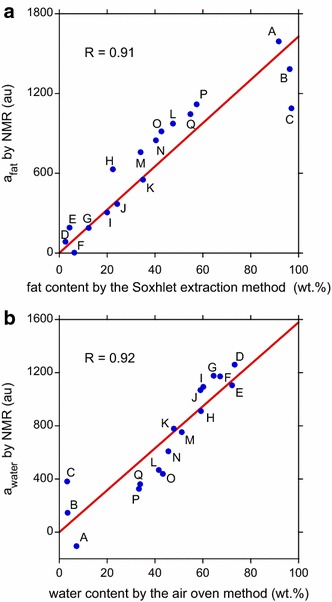
Results of the PAPS CPMG measurements of 17 beef block samples. The sample name and correlation coefficient, *R*, are indicated. Number of signal stacking = 4. **a** Calibration line for fat. **b** Calibration line for water

Equation (1) was fitted to the PAPS CPMG data of the 17 samples using the least squares method. The fitted *a*_fat_ and *a*_water_ values were combined with the conventional chemical analysis results to draw calibration lines for the number of signal stacking = 4 (Fig. 5). The obtained correlation coefficients, 0.91 and 0.92, are nearly equal to unity and acceptable because the values are similar to those obtained by previous studies using single-sided magnets [9, 12]. Although figures are omitted, the similar reasonable correlation coefficients were also obtained for the increased number of signal stacking, specifically 8, 16, and 32 (e.g., 0.92 for fat and 0.91 for water, number of signal stacking = 32).

**Fig. 6:**
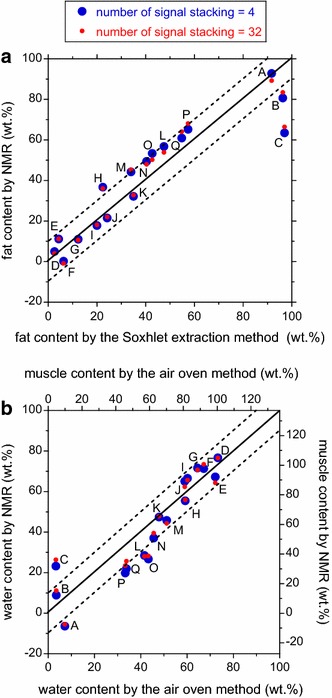
Cross plot of the fat and water content measured by NMR and by conventional analytical methods. The sample name is indicated. Three error contours, corresponding to 0 and ±10 wt%, are indicated by *solid * and *dotted lines*, respectively. **a** Fat content. **b** Water content. The water content was also converted into the muscle (i.e., water plus protein) content according to Fig. ESM_9

The calibration lines of Fig. 5 were used to convert the *a*_fat_ and *a*_water_ values into fat and water content, respectively (Fig. 6). Thanks to the high correlation coefficients shown in Fig. 5, it can be said that conventional chemical analysis and our NMR method show reasonable agreement. For the number of signal stacking = 4, the root mean square values of the error were 11.3 and 9.9 wt% for fat and water, respectively. These values were very slightly improved to 10.5 and 9.7 wt%, respectively, for the number of signal stacking = 32.

When discussing a marbling score, it is desirable to measure the muscle (water plus protein) content rather than the water content alone. Unfortunately, protein contains C, N, O, and S, which cannot be detected by proton NMR. Thus, it is difficult to directly quantify the muscle content using our proton NMR apparatus. However, by cross-plotting the fat and water content, it is possible to estimate muscle content using the procedure described in Fig. ESM_9. The estimated muscle content converted from the water content is shown in Fig. 6b.

## Discussion

Due to the well-designed RF coil and large magnet size, the sensed region is as large as 19 × 19 × 16 mm^3^ and has a large *B*_*z*_ value of 97 mT (Fig. 3). This enables us to perform measurements with reasonable S/N ratios at a small number of signal stacking (Fig. 4). The center of the sensed region is located 30 mm from the RF coil surface (Fig. 3b), thereby allowing in vivo measurements of the trapezius muscle, which is located at about 30 mm beneath the cattle’s skin.

Figure 3 shows that in reality the NMR signals come from a volume slightly larger than 19 × 19 × 16 mm^3^. However, the anatomical extent of the trapezius muscle of live cattle is much larger as follows. As for the *z*-direction, the normalized signal intensity is almost 0 and 100 % at 15 and 45 mm from the RF coil, respectively (Fig. 3b). On the other hand, the trapezius muscle of live cattle could extend from ≈10 to ≈50 mm beneath the cattle’s skin [30]. Thus, all the NMR signals would come from the trapezius muscle only, and the undesirable signal contamination from the adjacent tissues (subcutaneous fat and intermuscular fat) would be negligible. Anatomically the trapezius muscle extends much longer in the *x*- and *y*-directions than in the *z*-direction. Thus, the signal contamination cannot be a serious problem for the *x*- and *y*-direction. In conclusion, the NMR signal contamination from the outside of the trapezius muscle is negligible for our apparatus.

The root mean square values of the error were as small as about 10 wt% for the prototype (Fig. 6), which would be acceptable as a pilot device for use with live cattle. Figure 6 shows that accuracy levels are not significantly different between 4-fold stacking and 32-fold stacking, thus indicating that 4-fold stacking is sufficient for marbling measurements. Because the PAPS CPMG repetition time was 3 s, a 4-fold stacking experiment with an echo spacing of 0.5 ms and 1200 echoes can be performed with a total acquisition time as short as 3 × (4 − 1) + 0.0005 × 1200 ≈ 10 s. Since no sedative/anesthetic would be needed to ensure that animals remain steady during these ten-second-long NMR measurements, it can be said that our NMR apparatus does not impose serious loads on the cattle.

It should be noted that even though our magnet (Fig. ESM_1) is larger and heavier than the single-sided magnets used in previous studies [8–12] to increase the available investigation depth, it only weighs approximately 43 kg, and thus would be much smaller and lighter than magnetic circuits designed for whole-body magnetic resonance imaging of live cattle [23]. Furthermore, as can be seen in Fig. 1, our apparatus is portable such that it can be transported with ease from pasture to pasture and from auction to auction, thereby rendering excellent utility and cost performance.

The results of our experiments show that neither the accuracy nor the correlation coefficient was significantly improved when the number of signal stacking was increased from 4 to 32 (Figs. 5, 6). This suggests that the NMR data point scatter is not due to random noise, such as the RF noise leaked from the NMR transmitter. One possible cause is that *T*2_fat_ and *T*2_water_ in Eq. (1) somewhat differ from breed to breed and from age to age. This hypothesis could be tested by measuring *T2*_fat_ of the subcutaneous fat and *T*2_water_ of the round portion for many live cattle using our apparatus and by statistically examining the dependence of *T*2_fat_ and *T*2_water_ on the breed and age. If the dependence is revealed, *T2*_fat_ and *T2*_water_ in Eq. (1) should be determined appropriately based on the statistical data.

In relation to the current study, the following points should be noted:

1. The parameters, *T*2_fat_ and *T*2_water_, in Eq. (1) are effective *T*2 values that are weighted by the molecular diffusion and depend on the echo spacing [6]. Thus, the parameters should be reevaluated when using an echo spacing different from 0.5 ms. Several previous studies have remarked on the slight *T*2 change that occurs during the conversion of muscle to meat [21, 24]. Thus, *T*2_water_ in Eq. (1) also should be reevaluated by in vivo measurements when our measurement method is applied to live cattle. This reevaluation of *T*2_fat_ and *T*2_water_ would yield the change in the slopes of the calibration lines in Fig. 5.
2. Table 1 shows that, like the *T*2 measurement, there is a significant *T*1 difference between fat (Sample A) and water in lean meat (Sample E). This suggests that *T*1 relaxometry [7] is also applicable to the quantitative discrimination of fat and muscle. The *T*1 dependence on the strengths of the static magnetic field measured by fast field cycling (FFC) NMR [25] is shown in Fig. ESM_10 to provide helpful information about the magnet design as applied to *T*1 relaxometry.
3. Diffusometry is also possible using our apparatus because a small but non-zero field gradient survives in the sweet spot [22]. Two-dimensional NMR using diffusometry coupled with *T*2 relaxometry [6] would increase the accuracy of the quantification of water and fat molecules having different self-diffusivities (Table 1). However, two-dimensional NMR requires much longer time for the data acquisition compared with the simple *T*2 relaxometry performed in the present study. Use of sedative/anesthetic would be inevitable to ensure that cattle remain steady during the long-time two-dimensional NMR measurements, which is against the animal welfare. Thus, this tradeoff should be carefully discussed when measuring live cattle.
4. Currently, marbling scores are determined using the longissimus (rib eye) muscle, which is located about 100 mm beneath the cattle’s skin. The investigation depth of our prototype (i.e., 30 mm) could be lengthened to 100 mm if a larger permanent magnet with the same shape as Fig. ESM_1 is produced. However, it would be as heavy as 43 × (100/30)^3^ ≈ 1600 kg. It is very expensive and unrealistic to construct such a heavy permanent magnet. One possible solution for increasing investigation depth in the future might be the use of small high-temperature superconducting magnets that are currently under development [26].
5. This prototype can be used for diagnosis of cattle diseases, in addition to marbling measurements, because it is well-known that *T*1 and *T*2 values are different between normal and abnormal tissues [27]. For example, the diagnosis of muscular diseases [28, 30, 32] for live cattle would be made possible by in vivo relaxometry using the NMR surface scanner.

## Conclusions

We developed a prototype of an NMR surface scanner to quantify the water and fat in the trapezius muscle of live cattle. The scanner includes an original single-sided permanent magnet with depth of investigation of 30 mm and a sensed region as compact as 19 × 19 × 16 mm^3^. Using our apparatus, 17 beef meat block samples were measured successfully via NMR *T2* relaxometry with fourfold signal stacking, which yielded an measurement error of as small as approximately 10 wt%. This prototype would enable the in vivo marbling assessment of live cattle.

## Electronic supplementary material

Below is the link to the electronic supplementary material.

Supplementary material 1 (PDF 1745 kb)
