# Supplementary material for: Development of a Single-Sided Nuclear Magnetic Resonance Scanner for the In Vivo Quantification of Live Cattle Marbling
Source: Appl Magn Reson. 2015 Mar 18;46(5):593–606. doi: 10.1007/s00723-015-0657-4 (PMC4409640; doi:10.1007/s00723-015-0657-4)
Supplement: Supplementary file 1 — Supplementary material 1 (PDF 1745 kb) [file 723_2015_657_MOESM1_ESM.pdf]

Electronic Supplementary Material

Article title: Development of a Single-Sided Nuclear Magnetic Resonance Scanner for the *In Vivo* Quantification of Live Cattle Marbling

Journal name: Applied Magnetic Resonance

Author: Yoshito Nakashima

Address: National Institute of Advanced Industrial Science and Technology (AIST), Central 7, 1-1-1 Higashi, Tsukuba, Ibaraki 305-8567, Japan

E-mail: nakashima.yoshito@aist.go.jp

Contents

A. Magnet design (Fig. ESM_1) 2-3
B. RF coil design (Fig. ESM_2) 4
C. PAPS sequence (Figs. ESM_3to4) 5-6
D. Experiments using silicon rubber sheets (Fig. ESM_5) 7
E. List of beef block samples measured (Table ESM_1) 8
F. Experiments for beef block samples (Figs. ESM_6to8) 9-11
G. Water fraction to muscle fraction conversion (Fig. ESM_9) 12
H. *T1* dependence on the Larmor frequency (Fig. ESM_10) 13
I. References 14

A. Magnet design

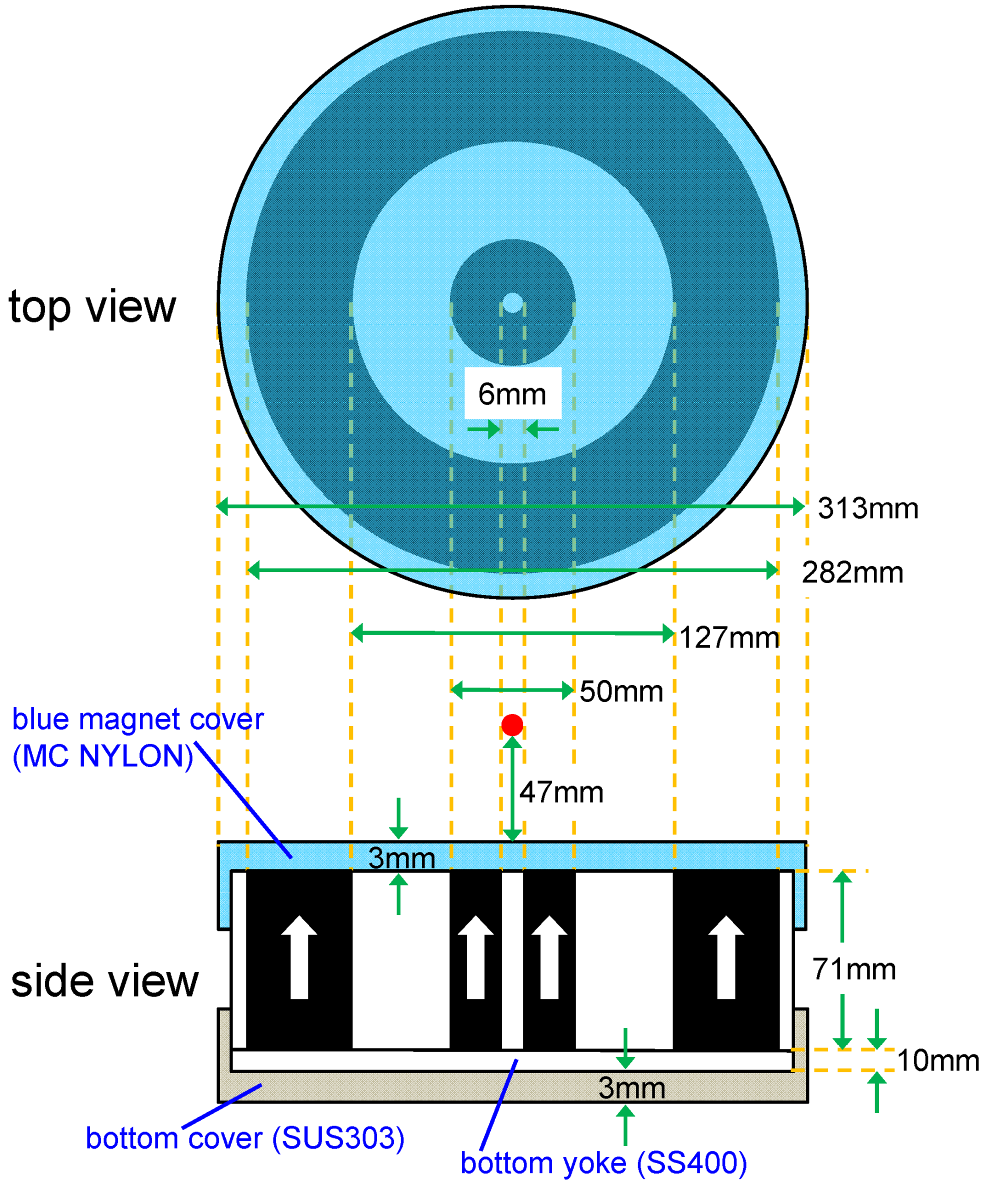

Fig. ESM_1 Brief structural overview of our axisymmetric single-sided magnetic circuit having a sweet spot. A large region with a homogeneous magnetic field (sweet spot) is generated by the two cylindrical Nd-Fe-B magnets (black) with the same direction of magnetization (indicated by open arrows). The center of the sensed region (indicated by the red dot) is 47 mm above the surface of the

blue magnet cover (MC NYLON), which is also the origin of the *x*-*y*-*z* coordinate system shown in Fig. 1b. The Nd-Fe-B magnet was made by Hitachi Metals, Ltd. (Tokyo, Japan). The residual flux density of the permanent magnet is 1.3 T, the coercivity is 1000 kA/m, and the reversible temperature coefficient is −0.11 %/ºC (the product name is NEOMAX-44H). The total weight of the magnetic circuit is approximately 43 kg. Although omitted in Fig. ESM_1 for simplicity, metallic frames made of aluminium (A6061) are installed to fix the relative position of the two concentric magnets. The bottom yoke and bottom cover are also metallic, and MC NYLON is a durable plastic. Thus, the mechanical stability of the magnetic circuit is ensured. The distance to the red dot from the surface of the NEOMAX-44H is 47 + 3 = 50 mm, and the aspect ratio divided by the overall diameter of the magnet (282 mm) is as large as 50/282 = 0.18, which is reasonable for a well-designed sweet-spot magnet [R1]. See refs [R2-R7] for details about the structure of the sweet-spot magnets related to our research

B. RF coil design

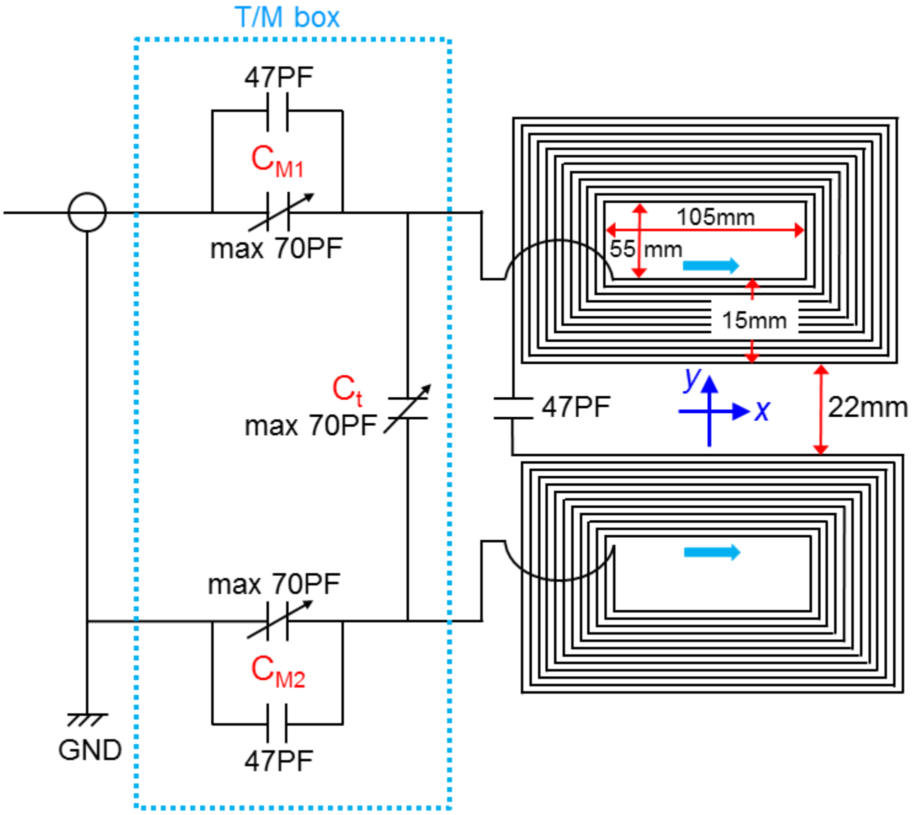

Fig. ESM_2 Schematics of the in house-built RF circuit including the plane coil and T/M box. The coil consists of two identical subunits (each 12-turn, line thickness 1.2 mm) divided by a chip capacitor. The RF current flows in the direction shown by the light blue arrows at the coil center in order to generate magnetic fields parallel to the face of the coil. The circuit is tuned using a trimmer capacitor (C_t) and matched using two trimmer capacitors (C_{M1} and C_{M2}) sample by sample. All capacitors are non-magnetic products produced by Voltronics Co. (Salisbury, MD). The approximate position of the *x*-*y* coordinate system of Fig. 1b is indicated

C. PAPS sequence

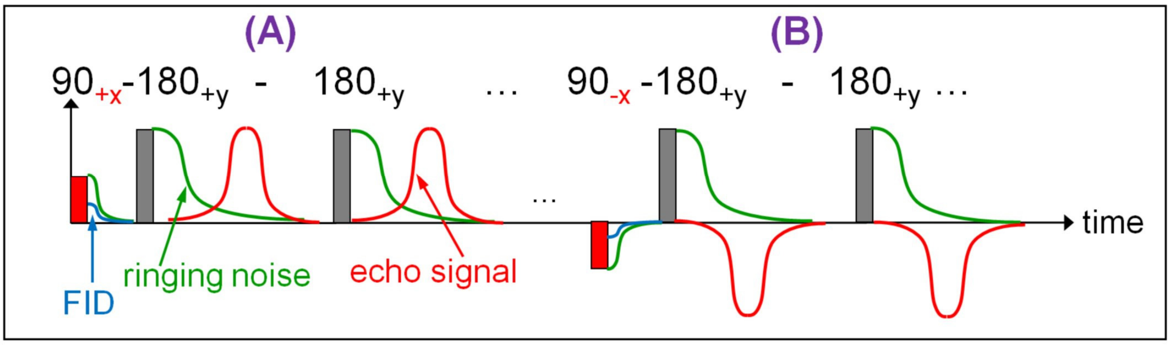

Fig. ESM_3 Pulse sequence for the phase-alternated pair stacking (PAPS) CPMG method. While only (A) is employed for the conventional CPMG, a pair of (A) and (B) is acquired for the PAPS CPMG followed by the calculation of ((A) – (B))/2. This calculation enables cancelation of the ringing noise (coherent noise) derived from the mechanical vibration of the RF coil for all echoes except the first free induction decay (FID) signal

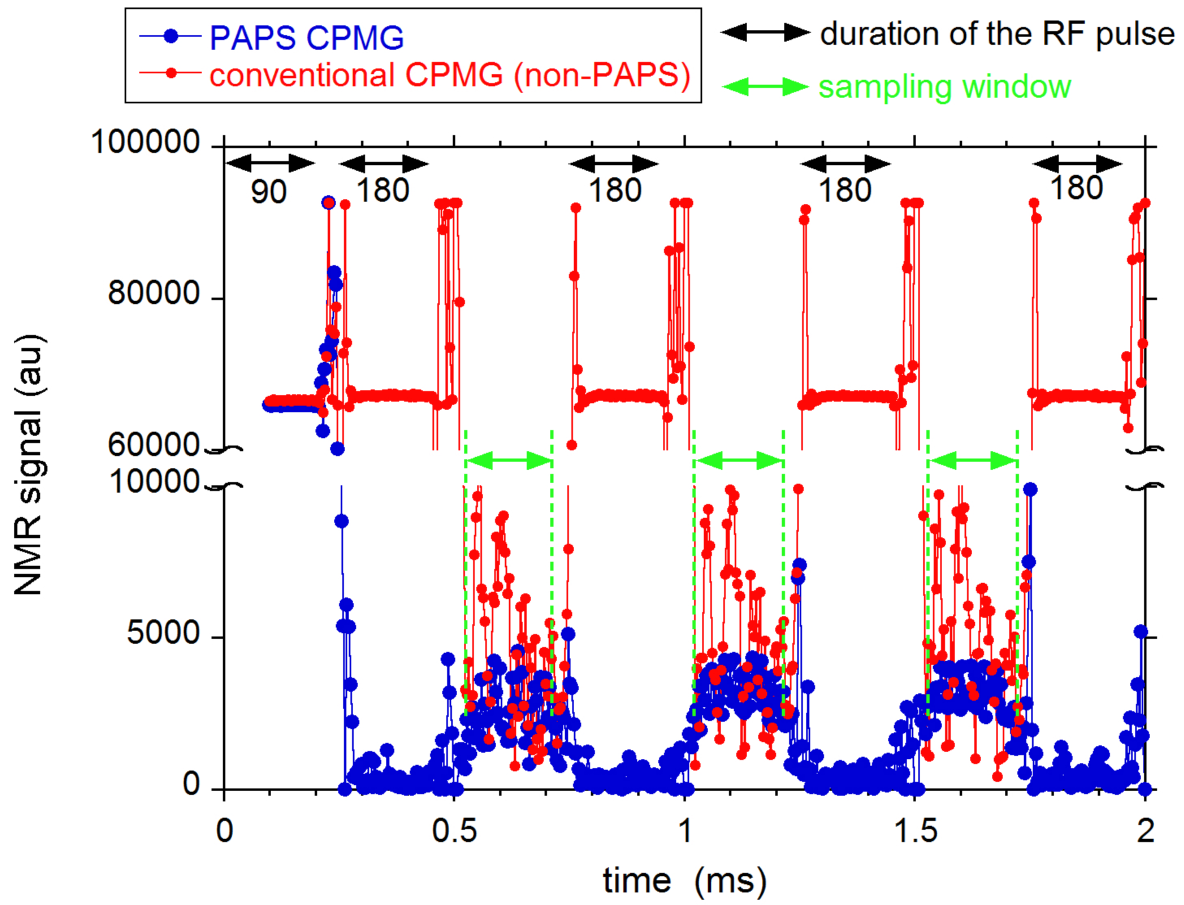

Fig. ESM_4 Example of raw time-series data detected by the RF coil for Sample M. The signal magnitude (i.e., root of the sum of the square of the in-phase component and that of the quadrature component) stacked over eight scans (i.e., four PAPS pairs) is shown as a function of time. The vertical axis is omitted for the interval of 10000 to 60000. The data acquisition by an AD convertor starts at 0.1 ms after the beginning of the 90º RF pulse. The echo spacing is 0.5 ms, the duration of the 90º and 180º RF pulses is 0.2 ms, the sequence repetition time is 3000ms, the total number of the echoes is 1200, and the length of the sampling window for averaging echo signals is 0.195 ms. The first three echoes can be seen at around 0.65, 1.15, and 1.65 ms. While large ringing noise survives in the sampling window for the non-PAPS sequence, the noise is successfully reduced for the PAPS sequence, thus allowing us to see the three echoes clearly. The specific absorption rate (SAR) for the RF coil was calculated to estimate the undesirable heating of the sample. As for the pulse sequence of Fig. ESM_4, the duty cycle is 0.08 and the magnitude of the RF magnetic field is 6×10^{-5} T. The radius of the spherically approximated sensed region is 0.01 m, the sample density is 1000 kg/m^3, and the electric conductivity of the meat is taken to be 0.5 S/m. Substituting these parameters into a well-known formula for the SAR calculation [R15], we obtained a SAR value of $\approx10^{-5}$ W/kg, which is much smaller than the regulation value of $\approx$1 W/kg. Thus, the RF heating is negligible for our apparatus

D. Experiments using silicon rubber sheets

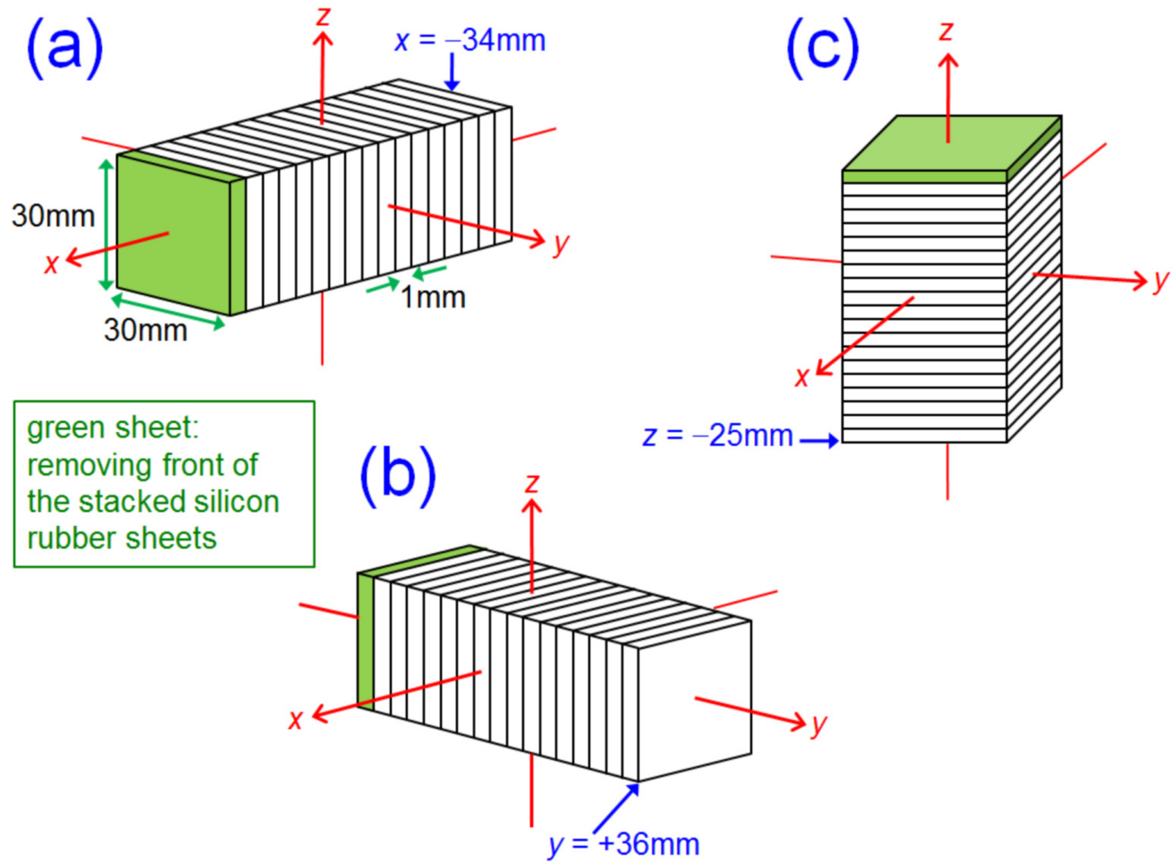

Fig. ESM_5 Stacking directions of silicon rubber sheets employed in the experiments for probing the sensitivity in the (a) *x*-direction, (b) *y*-direction, and (c) *z*-direction. The origin of the *x*-*y*-*z* coordinate system is located 47 mm above the blue magnet cover (Fig. 1b). NMR properties of the silicon rubber used ($T1$ = 229 ms, $T2$ = 86 ms, and the self-diffusivity = 2×10^{-11} m^2/s at 28ºC) were similar to those for the beef samples (Table 1), and the dimension was 30×30×1 mm^3 for each sheet. First, 70, 70, and 50 stacked sheets were placed in (a), (b), and (c), respectively, to completely cover the sensed region. Then the NMR signal intensity was measured by the method of the summation of echoes [R8-R9] at room temperature using the PAPS CPMG sequence. The parameters of the sequence were as follows: duration of the 90º and 180º pulses, 0.2 ms; echo spacing, 0.5 ms; A/D convertor sampling rate, 0.005 ms; sampling window length, 0.195 ms; repetition time of the sequence, 900 ms; number of echoes summed, 157; number of signal stacking, 90. The rubber sheets were removed sheet by sheet to measure the NMR signal intensity decrease that occurs as the number of sheets decreases. The front position of the removal is shown in green. The removal process ends when the removing front reaches $x = -34$ mm in (a), $y = +36$ mm in (b), and $z = -25$ mm in (c). To show the symmetry-derived data agreement between (a) and (b), the sign of the *y*-coordinate in Fig. 3(a) was taken to be negative (i.e., $-y$)

E. List of beef block samples measured

Table ESM_1 List of beef meat block samples. ID numbers are assigned to all cattle raised in Japan by the National Livestock Breeding Center (https://www.id.nlbc.go.jp/english/). Detailed information (e.g., breed, date of birth, and gender) is available at the URL

sample	portion	breed	ID	raised in
A	kidney fat (suet)	Japanese Black	1376937624	Japan
B	kidney fat (suet)	Japanese Black	1344006079	Japan
C	kidney fat (suet)	Japanese Black	1351210186	Japan
D	round (lean meat)	N/A	N/A	Australia
E	round (lean meat)	N/A	N/A	Australia
F	round (lean meat)	N/A	N/A	Australia
G	round	Crossbreeds	1362105907	Japan
H	round	Japanese Black	1338663271	Japan
I	round	Japanese Black	1351210186	Japan
J	tenderloin	Japanese Black	1376937624	Japan
K	tenderloin	Japanese Black	1252540108	Japan
L	rib (trapezius muscle)	Japanese Black	1376937624	Japan
M	rib (trapezius muscle)	Japanese Black	1344006079	Japan
N	rib (latissimus dorsi muscle)	Japanese Black	1344006079	Japan
O	sirloin (longissimus muscle)	Japanese Black	1344006079	Japan
P	rib (longissimus muscle)	Japanese Black	1351210186	Japan
Q	rib (semispinalis dorsi muscle)	Japanese Black	1351210186	Japan

F. Experiments for beef block samples

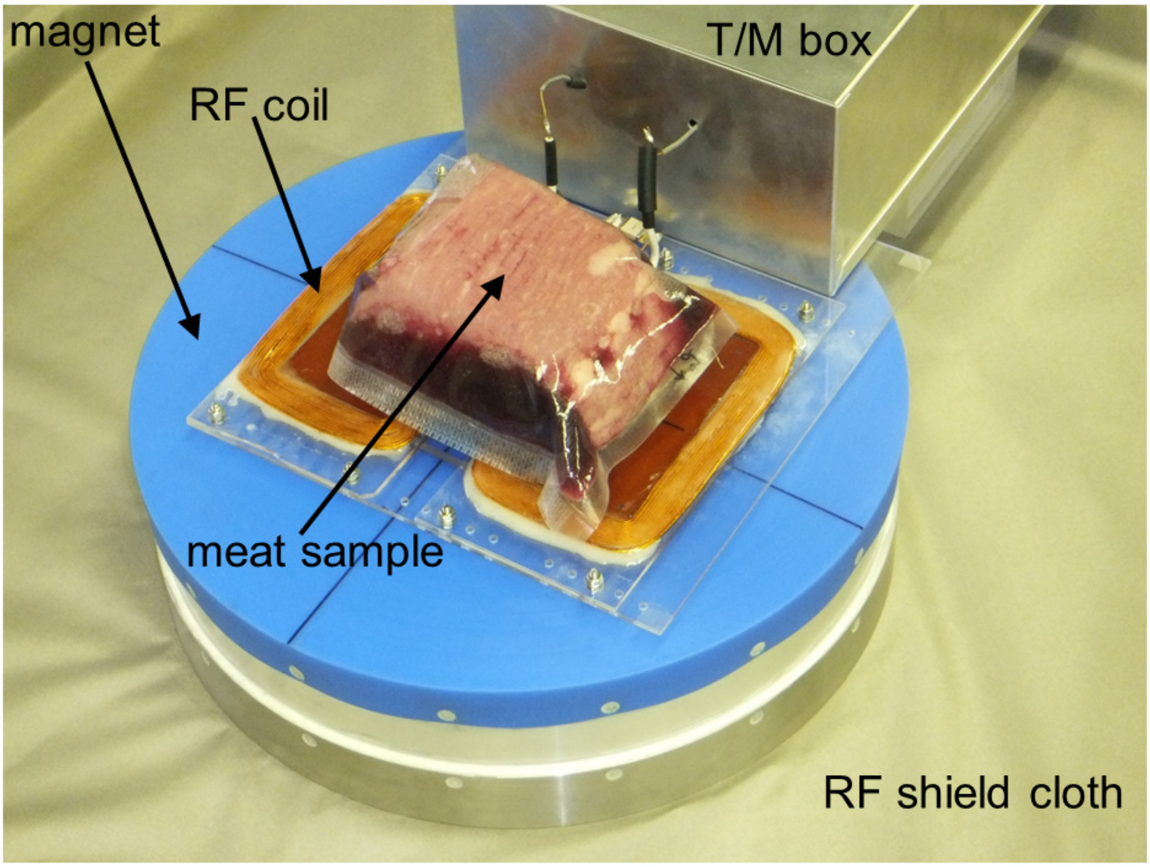

Fig. ESM_6 Packed beef meat block (Sample H) placed on the RF coil. Although omitted in this photo, in actual experiments, a copper foil (300×300×0.2 mm^3) with slits was inserted between the RF coil and the blue magnet cover to reduce the undesirable eddy currents induced by the RF pulses

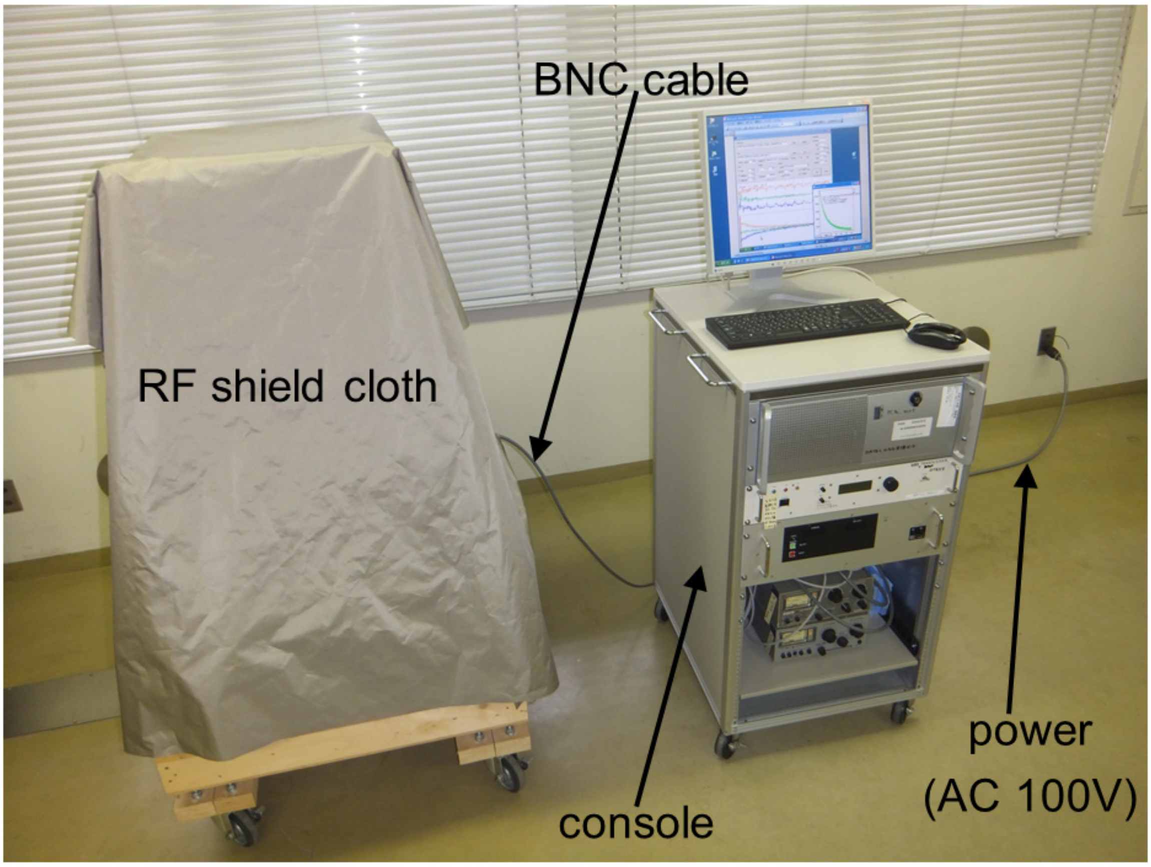

Fig. ESM_7 Packed beef meat sample shown in Fig. ESM_6 completely covered with an RF shield cloth (product name MS-PY, about −80 dB) produced by Microwave Absorbers Inc. (Tokyo, Japan). This cloth was used during all NMR measurements to reduce the electromagnetic noise. We confirmed that this shielded tent successfully reduced the noise level by about 50% compared with the case without it

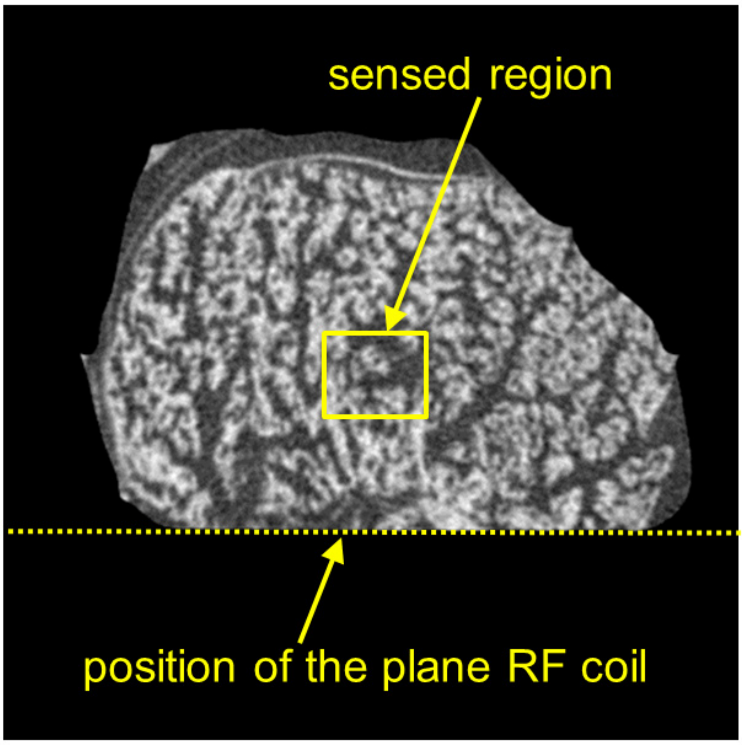

Fig. ESM_8 Two-dimensional X-ray CT image of the packed beef meat sample P (vertical cross-section) obtained by a medical CT scanner at GSJ-Lab, AIST [R10]. The image dimension was 448^2 voxel = 140^2 mm^2, the slice thickness was 1 mm, and the acceleration voltage of the tube was 100 kV. Here, due to the chemical and density differences, the fat and muscle are clearly distinguishable (dark and bright in the CT image, respectively [R11]). The supposed position of the plane RF coil (i.e., $z = -30$ mm) and sensed region (19×16 mm^2) are superimposed

G. Water fraction to muscle fraction conversion

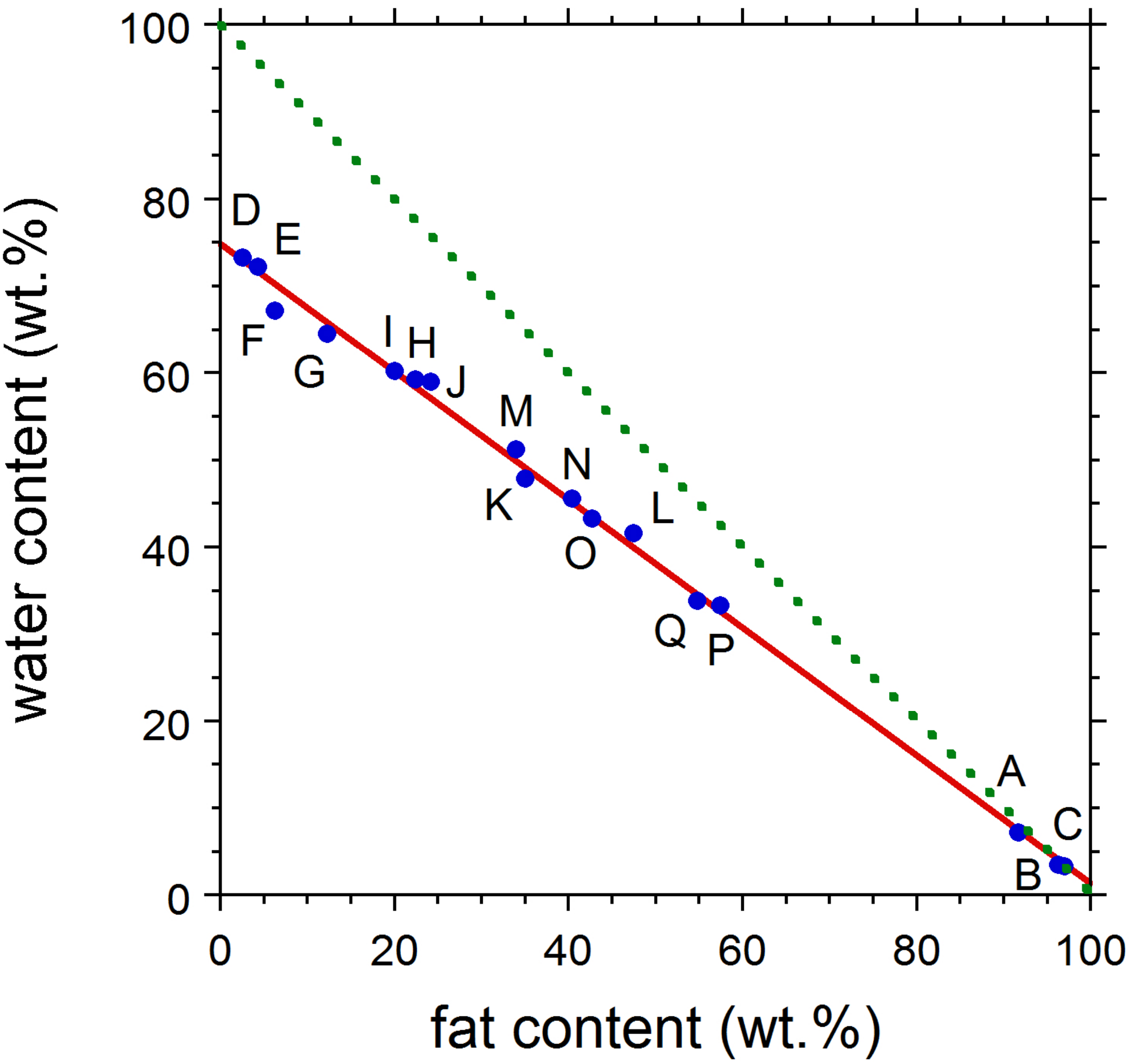

Fig. ESM_9 Cross plot of fat and water in the 17 beef meat block samples. The sample name is indicated. The values for the fat and water were obtained by the conventional Soxhlet extraction method and air oven method, respectively. The slope of the fitted line in red is −0.73 (not −1). The primary reason why the data points do not obey the green dotted line with a slope of −1 is that meat contains protein as well as water. Using the obtained slope of −0.73, it is possible to estimate the content of muscle (i.e., water plus protein) by the following formula: (muscle content in wt.%) = (water content in wt.%)/0.73. The protein content for Sample H was measured by the conventional Kjeldahl method to obtain 18.0 wt.% (water 59.0 wt.%, fat 21.8 wt.%, ash 0.9 wt.%, carbohydrate 0.3 wt.%, and total 100 wt.%). The obtained result indicates that (water)/(muscle content) = (water)/(water plus protein) = 59/(59 + 18) = 0.77. This value agrees well with the fitted slope value of 0.73, thus supporting the validity of the formula. The water content is converted into the muscle content in the axes of Fig. 6b using this formula

H. *T1* dependence on the Larmor frequency

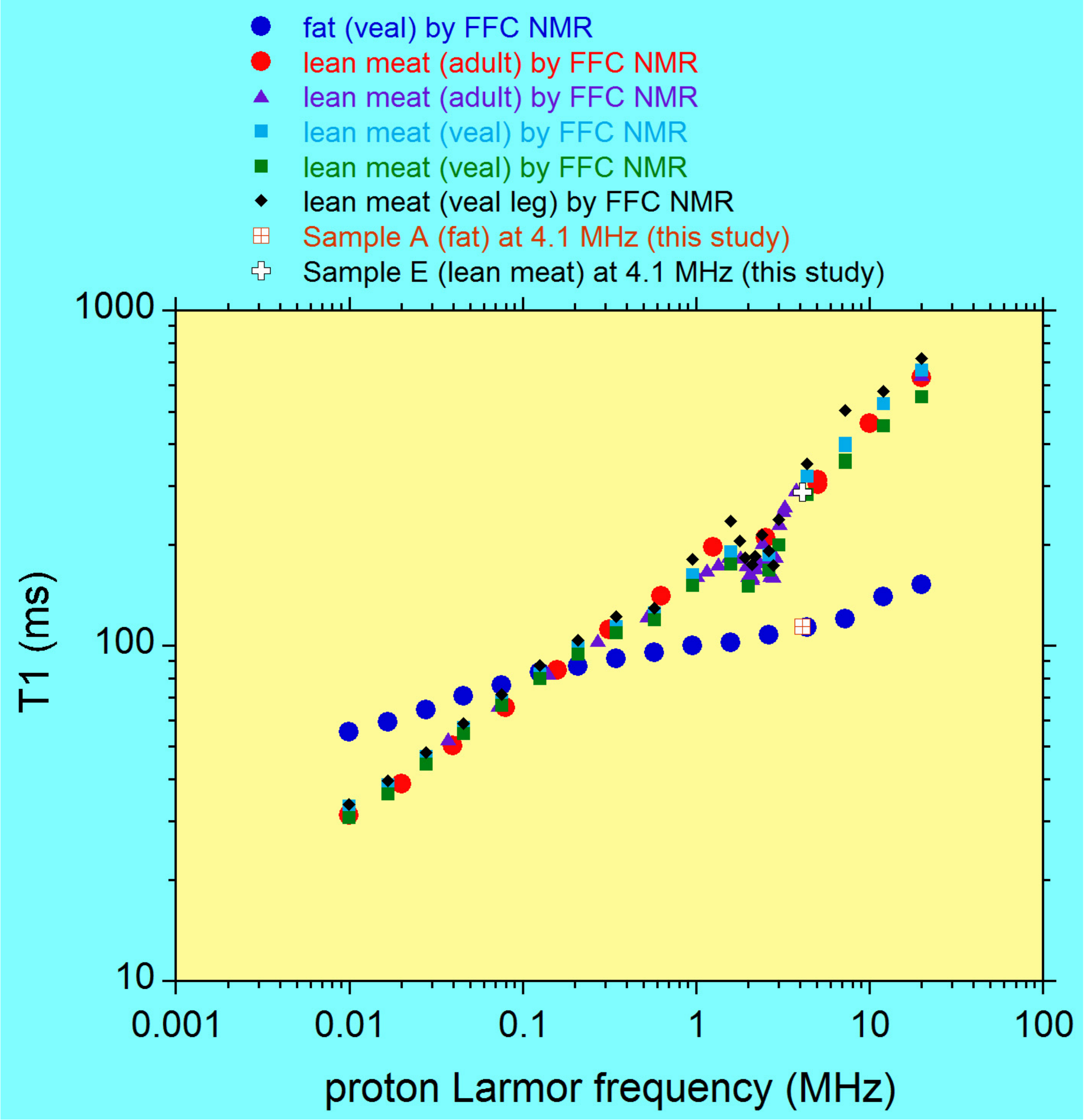

Fig. ESM_10 *T1* values of water molecules in lean meat and of fat molecules for various static magnetic field strengths (the field strength is converted into the Larmor frequency of protons). Beef meat data measured by FFC NMR at 40ºC basically follow ref [R12], but a veal leg data set was newly added. Two data points measured at 4.1 MHz and 39ºC are added from Table 1, showing the reasonable agreement with ref [R12]. In terms of the discrimination of muscle and fat, the *T1* contrast between water and fat should be larger. There is a crossover at around 0.1 MHz, and anomalous dips of water *T1* values occur at 2.13 and 2.81 MHz due to the cross-relaxation of ^{1}H with ^{14}N in the amide groups [R13-R14]. Since these interfere with *T1* relaxometry discrimination, low-field NMR is not recommended. In conclusion, in terms of *T1* relaxometry, a strong magnetic field larger than about 4 MHz is desirable because it ensures a stronger *T1* contrast between water and fat

I. References

R1. E. Fukushima, Abstract of the 8th International Conference on Magnetic Resonance Microscopy, Mibu, Japan (2005)

R2. E. Fukushima, Abstract for the 5th meeting of the NMR Microimaging Study Group, Tsukuba, Japan (2000)

R3. S. Utsuzawa, R. Kemmer, Y. Nakashima, Abstract of the 8th International Conference on Magnetic Resonance Microscopy, Mibu, Japan (2005)

R4. S. Utsuzawa, R. Kemmer, Y. Nakashima, K. Kose, Abstract of the 46th Experimental Nuclear Magnetic Resonance Conference, Rhode Island, USA (2005)

R5. S. Utsuzawa, E. Fukushima, Abstract of the 48th Experimental Nuclear Magnetic Resonance Conference, Florida, USA (2007)

R6. S. Utsuzawa, E. Fukushima, Abstract of the 9th International Conference on Magnetic Resonance Microscopy, Aachen, Germany (2007)

R7. S. Utsuzawa, E. Fukushima, Y. Nakashima, Abstract of the 9th International Bologna conferences of Magnetic Resonance in Porous Media. Cambridge, USA (2008)

R8. D. Allen, C. Flaum, T.S. Ramakrishnan, J. Bedford, K. Castelijns, D. Fairhurst, G. Gubelin, N. Heaton, C.C. Minh, M.A. Norville, M.R. Seim, T. Pritchard, R. Ramamoorthy, Oilfield Rev. **12**, 2-19 (2000)

R9. K.-J. Dunn, D.J. Bergman, G.A. LaTorraca, *Nuclear Magnetic Resonance Petrophysical and Logging Applications* (Pergamon, New York, 2002)

R10. Y. Nakashima, Eng. Geol. **56**, 11-17 (2000)

R11. T. Nade, K. Fijita, M. Fujii, M. Yoshida, T. Haryu, S. Misumi, T. Okumura, Anim. Sci. J. **76**, 513-517 (2005)

R12. Y. Nakashima, Japanese Patent (application number, 2007-043142) (2007)

R13. R. Kimmich, *NMR-Tomography, Diffusometry, Relaxometry* (Springer-Verlag, Berlin, 1997)

R14. R. Kimmich, E. Anoardo, Prog. Nucl. Magn. Reson. Spectrosc. **44**, 257-320 (2004)

R15. T. Kasai, T. Doi, (Eds.), *Imaging Technology for Magnetic Resonance* (in Japanese) (Ohmsha, Tokyo, 2008)
